# Challenges in Accessing and Delivering Maternal and Child Health Services during the COVID-19 Pandemic: A Cross-Sectional Rapid Survey from Six States of India

**DOI:** 10.3390/ijerph20021538

**Published:** 2023-01-14

**Authors:** Saurabh Sharma, Sumit Aggarwal, Ragini Kulkarni, Dinesh Kumar, Bijaya Kumar Mishra, Gaurav Raj Dwivedi, K. Rekha Devi, Raja Sriswan Mamidi, Khangembam Jitenkumar Singh, Lucky Singh, Damodar Sahu, Tulsi Adhikari, Saritha Nair, Anil Kumar, Atul Juneja, Anshita Sharma, Shahina Begum, Suchitra Surve, Ranjan Kumar Prusty, Surendra Kumar, J. J. Babu Geddam, Gargi Meur, Mahesh Kumar Mummadi, Uma Kailash, Subrata Kumar Palo, Srikanta Kanungo, Jaya Singh Kshatri, Ajit Kumar Behera, Swagatika Swain, Rajeev Singh, Kamran Zaman, Hirawati Deval, Ashok Kumar Pandey, Abu Sarkar, Rajni Kant, Kanwar Narain, Luigi D’Aquino, Asheber Gaym, Vivek Virendra Singh, M. Vishnu Vardhana Rao

**Affiliations:** 1 National Institute of Medical Statistics, Ansari Nagar, New Delhi 110029, India; 2 Indian Council of Medical Research, Ansari Nagar, New Delhi 110029, India; 3 National Institute for Research in Reproductive Health, Mumbai 400012, India; 4 National Institute for Research in Tribal Health, Jabalpur 482003, India; 5 Regional Medical Research Centre, Bhubaneswar 786001, India; 6 Regional Medical Research Centre, Gorakhpur 273013, India; 7 Regional Medical Research Centre, NE Region, Dibrugarh 751023, India; 8 National Institute of Nutrition, Hyderabad 500007, India; 9 UNICEF, India Country Office, New Delhi 110003, India

**Keywords:** COVID-19, maternal health, child health, childhood immunisation, India

## Abstract

Background/Objectives: Globally, the COVID-19 pandemic and its prevention and control policies have impacted maternal and child health (MCH) services. This study documents the challenges faced by patients in accessing MCH services, and the experiences of health care providers in delivering those services during the COVID-19 outbreak, explicitly focusing on the lockdown period in India. Methods: A cross-sectional study (rapid survey) was conducted in 18 districts from 6 states of India during March to June, 2020. The sample size included 540 MCH patients, 18 gynaecologists, 18 paediatricians, 18 district immunisation officers and 108 frontline health workers. Bivariate analysis and multivariable analysis were used to assess the association between sociodemographic characteristics, and challenges faced by the patients. Results: More than one-third of patients (n = 212; 39%) reported that accessing MCH services was a challenge during the lockdown period, with major challenges being transportation-related difficulties (n = 99; 46%) unavailability of hospital-based services (n = 54; 23%) and interrupted outreach health services (n = 39; 18.4%). The supply-side challenges mainly included lack of infrastructural preparedness for outbreak situations, and a shortage of human resources. Conclusions/Recommendations: A holistic approach is required that focuses on both preparedness and response to the outbreak, as well reassignment and reinforcement of health care professionals to continue catering to and maintaining essential MCH services during the pandemic.

## 1. Background

COVID-19, which was declared a pandemic in March 2020 by the WHO [1], has disrupted health care services in both developed and developing countries, and as of May 30, 2022, more than six million people have died, and the numbers are still rising [2]. Since the Spanish flu, COVID-19 is one of the largest public health crises the world has witnessed. The indirect impact of COVID-19 on routine maternal and child health services has been documented from many LMIC (low- and middle-income countries) [3,4,5]. Countries opted for various stringent control measures, including social distancing, containment strategies, lockdown, and restricted inter-country and inter-state travel. Prioritizing only essential health care services was projected to indirectly impact the delivery of routine indispensable services, such as maternal and child health (MCH) care services.

The WHO had developed guidelines to support countries’ preparedness and response to the COVID-19 pandemic, prioritizing continuing maternal and child health care and immunisation services during the pandemic [1]. Studies have documented the impact of COVID-19 on maternal and child health outcomes from developed countries, and have shown that COVID-19 adversely affected maternal and perinatal mortality [6,7,8]; the impact is seen even more in LMICs, where the health infrastructure is already overburdened [5,9,10,11,12,13,14].

The challenges faced by both the patients in accessing MCH care, and the difficulties experienced by the health care providers in delivering quality care, have been documented in many studies [15,16,17,18]. The preparedness, redistribution, and reinforcement of health care professionals and medical equipment to contain the pandemic led to conversions of health care facilities into COVID-19 treatment facilities and quarantine and isolation centres, which further compounded inaccessibility to routine health care [16,19,20].

India also implemented the global strategy to contain the spread of the virus by imposing a countrywide lockdown for four months, beginning March 2020, which led to interruption of hospital-based and outreach services [21,22]. In parallel, the Ministry of Health and Family Welfare, India (MOHFW), also released guidance notes on maintaining maternal and child health services during the pandemic, with specific guidelines released by the Indian Council of Medical Research on management of pregnant women during the COVID-19 outbreak [23].

The direct and indirect impacts of COVID-19 and its prevention policies on MCH services have been documented in the Indian context in several regional studies which primarily focused on utilisation of hospital-based services [21,22]. There is a continued need to understand the impact of lockdown restrictions on the utilisation of MCH services. This study aimed to document the challenges faced by patients in accessing MCH services, and experiences of health care providers in delivering MCH services during the COVID-19 outbreak, explicitly focusing on the lockdown period.

## 2. Methods

### 2.1. Study Design and Setting

A cross-sectional study (rapid survey) was conducted in 18 districts from six states of India, in order to gather information about access to, and availability of, essential maternal and child health services among MCH patients and health care providers; the study focused specifically on the national lockdown in India, beginning from the last week of March 2020, to June 2020. Telephone interviews were conducted during the period from October 2020 to January 2021, using semi-structured questionnaires.

The country was categorised on the basis of six geographical regions, viz, North, South, East, West, North East, and Central; from each region, a state was selected, namely Maharashtra, Odisha, Assam, Uttar Pradesh, Madhya Pradesh and Telangana, respectively. The selection of districts was based on the Ministry of Health and Family Welfare (MOHFW) order, dated 30 April 2020, on COVID-19 categorisation of districts [24], as the study primarily focused on the lockdown duration. The districts in India were classified based on the COVID-19 caseload as red, orange and green zone districts, where red signifies a higher caseload.

Two red districts and one green zone district were randomly selected from each state to include eighteen districts. In each district, interviews were conducted using semi-structured questionnaires with district and block level health officials involved in planning and implementing MCH services, physicians and paramedical staff, including grass-root-level workers.

### 2.2. Sample Size for Patients

In view of the COVID-19 pandemic restrictions and inaccessibility during the conduct of this rapid assessment survey, non-probability sampling was considered with integration of key principles of sampling, i.e., systematic stratified random sampling. A minimum sample size of 30 MCH patients per district was considered, i.e., a total of 540 MCH patients from 18 districts.

### 2.3. Sample Size for Health Care Workers

The sample size for health care officials from each district (subdivision of a province) included gynaecologists n charge of MCH services (1), SNCU (Sick New Born Care Unit) paediatricians in charge (1), district immunisation officers (DIOs) (individuals who coordinated childhood immunisation activities in the district) (1). (i.e., a total of 18 gynaecologists, 18 paediatricians and 18 DIOs from 18 districts.)

The sample size of frontline health workers from each district included three auxiliary urse midwives (ANMs) and three accredited social health activist (ASHA) workers (i.e., a total of 54 ANMs and 54 ASHA workers from 18 districts).

### 2.4. Sampling Strategy

#### 2.4.1. Selection of MCH Patients

Pregnant women, lactating women or women with children aged six weeks to two years during March/April/May/June 2020, and women residing in the current address for the past year were included in the study. A total of 30 MCH patients were interviewed from each district; hence, there was a total of 90 from each selected state (30 per district, three districts in each state). Thus, a total of 540 patients were interviewed from six states. A line list of sub-centres under the selected Community Health Centre (CHC) was obtained from the district health authorities. For the selection of patients, five sub-centres under the one selected Community Health Centre (CHC) were randomly chosen from the line list. From these selected sub-centres, ANMs, i.e., front line workers, were contacted, and a list of patients was requested with their contact numbers. A total of 6 patients (assuming 40% non-response) from each sub-centre were randomly selected from the list except for the state of Telangana, where the investigators used the KCR (K Chandrashekar Rao) kit district-wise digital database of MCH patients to obtain the contact details of the patients.

#### 2.4.2. Selection of Health Care Workers

**Figure 1:**
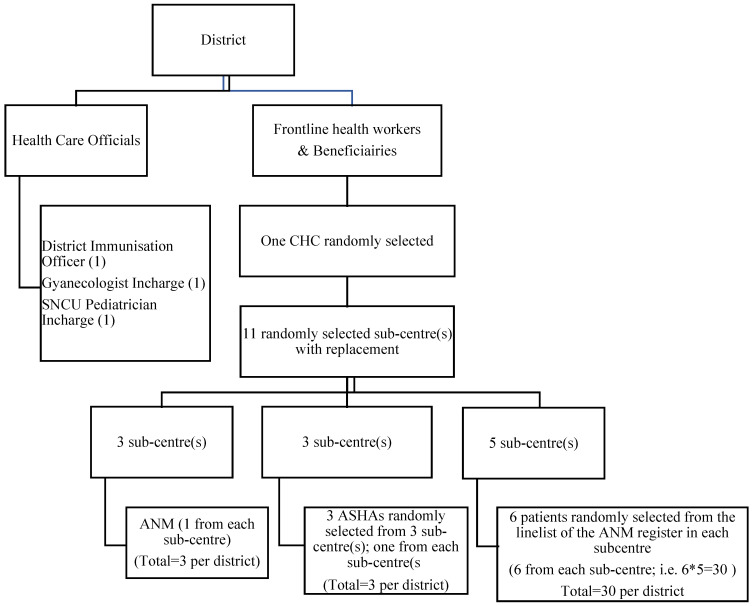
Flow diagram depicting the sampling strategy of HCW and patients for each district. SNCU—Sick Newborn Care Unit; CHC—Community Health Centre; ANM—auxiliary urse Midwife; mASHA—accreditated social health activist.

The selection strategy used is graphically presented via a flow diagram (Figure 1). From each selected district, health officials and frontline health care workers were interviewed. District immunisation officers (DIO) (1); gynaecologist/obstetrician in charge in the district hospital; SNCU (Special Newborn Care Unit) paediatrician in charge (1) and frontline health workers (i.e., three randomly selected ASHA workers and three ANMs). A line list containing the list of sub-centres with the name and contact details of the ANM/ASHA worker posted under the sub-centre was obtained from district health authorities, and three sub-centres were randomly selected from the list. From these three sub-centres, three ANMs were randomly selected, i.e., one from each sub-centre. Similarly, three ASHA workers from three randomly selected sub-centres were randomly chosen, other than the ones selected for the ANMs. Thus, a total of six frontline health care workers (i.e., three ANMs and three ASHA workers) were selected from six different sub-centres (one from each sub-centre).

### 2.5. Data Collection

Contact details of the health care providers were sought from the district health authorities after prior approvals from the district chief edical officer. The health care providers were initially contacted by the site investigators and briefed about the study. Furthermore, two field investigators were recruited at each site and trained for conducting telephone interviews. The telephone interviews were recorded, and the verbatim was transcribed into hard copies. The interview schedule (structured questionnaire) of the health care workers primarily had sections on the impact of the first lockdown on MCH services, and the challenges faced in delivering these services.

The Interview schedule (semi-structured questionnaire) of the patients primarily focused on the health outcomes and health care experiences during the first lockdown of the COVID-19 outbreak. The open-ended question was related to the challenges faced by the patients in accessing MCH services. The average duration of the interviews was around 30–40 min.

### 2.6. Data Processing and Analysis

The data were entered in CSPro version 7.4. and were analysed using SPSS Version 21. Categorical data were analysed using descriptive statistics (frequency and percentage).

The present study had as its outcome variable, challenges faced during the COVID-19 first lockdown period for maternal and child health care utilisation. It was captured by asking the question “Did you face any challenges during the COVID-19 lockdown period in accessing MCH services”? The response was coded into Yes and No. If the response was Yes, then it was followed by an open-ended question which asked about the challenges faced during the lockdown period.

Relevant socioeconomic and demographic factors included in the analysis were the following: women’s age (15–24 and 25 years and above), education of women (no schooling and less than 5 years; 5–7 years, 8–9 years, 10–11 years, 12 or more years), number of children (no child, one, two, more than two), having a ration card (yes and no), main source of income (agriculture, kkilled/unskilled labour, salaried employment/professional, small business/pension/rent/dividend/others) and main source of income during lockdown and COVID-19 zone (red and green).

To identify factors associated with the challenges faced during the COVID-19 lockdown period for utilisation of maternal and child health care services, bivariate and multivariable analyses were performed. Bivariate analyses were performed to understand the nature of association between challenges faced during the COVID-19 lockdown in utilisation of maternal and child health care services by selected socioeconomic and demographic background characteristics. Multivariate logistic regression was used to investigate which factors best explained and predicted the challenges faced during the COVID-19 lockdown for utilisation of the health outcome. Two researchers independently read the responses of the open-ended question and identified the themes. The responses were then categorised into the identified themes. 

### 2.7. Ethical Considerations

The study was approved by the ICMR-Central Ethics committee on Health Research (NCDIR/BEU/ICMR-CECHR/75/2020), and each study site also availed independent approval from their Institutional Ethics Committee, namely the ICMR-NIMS Ethics Committee on Health research, ICMR-NIRRH Ethics Committee for Clinical Studies, ICMR-NIN Institutional Ethics Committee, ICMR-RMRC Institutional Ethics Committee Bhubaneswar, ICMR-RMRC Human Ethical Committee Gorakhpur, and ICMR-RMRC North East Region Institutional Ethics Committee. Verbal consent was obtained from all participants, which was recorded, and no incentive was paid for participation. The names and telephone numbers were not entered into the master data file, in order to maintain confidentiality and anonymity.

## 3. Results

A total of 540 MCH patients and 18 gynaecologists, 18 paediatricians, 17 district immunisation officers (one did not give consent) and 108 frontline health workers (i.e., 54 ASHA workers and 54 ANMs) were interviewed. The overall response rates for MCH patients and health care workers were 66% and 88% respectively.

**Figure 2:**
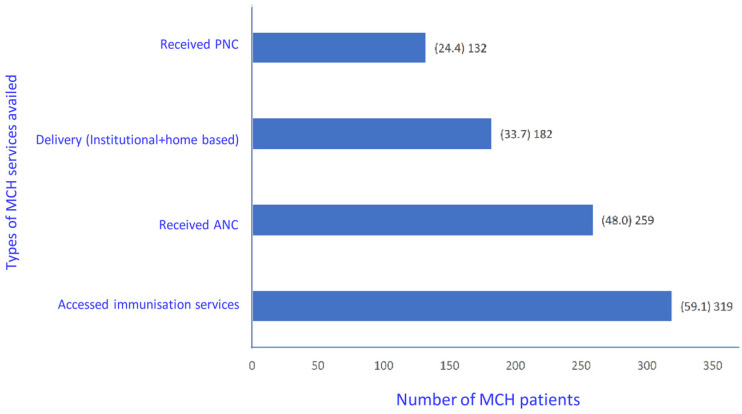
Distribution of patients based on the MCH services accessed in six states of India, March-June 2020 (n = 540). MCH Patients might be eligible for multiple services hence the sum is higher than 540. MCH—maternal and child health, ANC—antenatal care, PNC—postnatal care received by mother.

Due to the selection criteria that was followed, patients may have been eligible for accessing more than one service, i.e., antenatal services, delivery care services, postnatal care received by the mother, and immunisation services. The breakup of the patients based on the services availed is provided in Figure 2.

Table 1 shows the patients’ sociodemographic distribution regardless of the MCH service availed. About three-fifths of the women had received 10 or more years of education, and around half of the patients (47.4%) belonged to the 20–24 age group. More than one-fourth of the respondents’ main source of income was agriculture (28.9%), and more than half of the respondents (56.9%) reported that they had no income during the lockdown period (Table 1).

### 3.1. Challenges Faced by the Patients in Accessing MCH Services during the Lockdown Period

**Figure 3:**
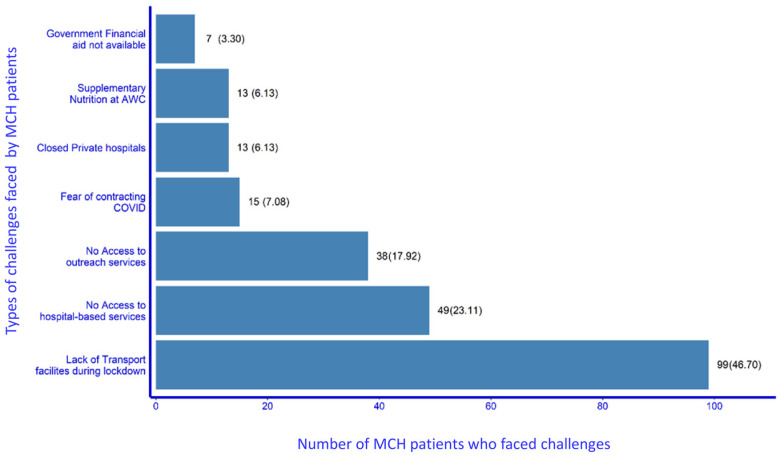
Types of challenges in accessing MCH services faced by patients during the COVID-19 lockdown (n = 212). Footnote: It is possible for one patient to report more than one challenge; hence, the sum is more than 212. AWC—Anganwadi Centre: government centre where supplementary nutrition and counselling is given to pregnant women, lactating mothers and children up to the age of six years.

Out of 540 patients, around three-fifth, 328 (61%), reported that they did not face any challenge in accessing MCH services during the lockdown period. Of the remaining 212 women, 99 (46.7%) reported transportation-related challenges, 49 (23%) women reported difficulty in accessing hospital-based services, while 38 (17.9%) reported interrupted outreach services. Fear of contracting COVID-19 was reported as a challenge by 15 (7%) of the patients (Figure 3).

The results of the bivariate analysis that examined the association between challenges and socio-demographic characteristics are shown in Table 2. The results of the multivariable analysis suggest (Table 2) that the odds of the challenges faced by women in the age group 25 and above were higher (OR = 1.57; 1.06–2.32) than those women belonging to the age group 15–24. Those women that had more than two children at the time of interview were 64 percent (OR = 0.36; 0.16–0.81) less likely to report challenges compared to those women who had no children.

The challenges were further elaborated and categorised into institutional and outreach services.

#### 3.1.1. Institutional Services

Unavailability of public transport to reach health facilities was one of the major challenges reported by women. During the lockdown period, public transport services were closed, hiring private vehicles was very costly and referral/ambulance services were also interrupted. Patients also reported that private health facilities were closed entirely during the lockdown, nor could they avail MCH services. In some areas, many of the public hospitals were converted into COVID-19-dedicated hospitals, resulting in patients being unable to avail services. Some of the challenges mentioned by the patients are quoted below:

*“We faced lots of problem during going to hospital and home as we could not find any vehicle for that. whatever we found they demanded more money from us.”*

(MCH Beneficiary No 59)

*“During Covid 19, there was no transport so my husband had to rent a private vehicle for my check-up.”*

(MCH Beneficiary No 75)

*“During the last moment of delivery, we didn’t get any ambulance services, we went to hospital by auto.”*

(MCH Beneficiary No 77)

#### 3.1.2. Outreach Services

The difficulties in accessing outreach services were related to interrupted services from ASHA/ANM (38; 17.9%), and the irregular supply of supplementary nutrition by the Anganwadi centres (AWCs—government-run centres providing supplementary nutrition services for pregnant females and children up to the age of 6 years) (13; 6.1%). Fear of contacting COVID-19 (15; 7%) was not only at the individual level, there was constant pressure both from family members and from the community at large.

### 3.2. Health Care Workers’ Experiences during the Lockdown Period of the COVID-19 Pandemic in India

#### 3.2.1. Institutional Services

The majority of the gynaecologists (15; 83.3%) reported a decline in both ANC OPD services, while half (9; 50%) of the respondents reported a decline in inpatient admissions. Conversion of district hospitals into dedicated COVID-19 hospitals was also reported by the gynaecologists. The majority (17; 94%) of paediatricians reported that admission rates in SNCUs were affected due to COVID-19. A downward trend in both inborn (14; 82.4%) and outborn SNCU (12; 70.6%) admissions was seen during the lockdown period. The primary reason was a decline in the number of deliveries conducted in the district hospitals, which led to a reduction in the number of inborn transfers. Furthermore, interrupted referrals from peripheral health facilities and frontline health workers were also mentioned as the reasons for the decline in outborn SNCU admissions. Out of the eighteen paediatricians interviewed, eleven (61.1%) reported allocation of separate units/isolation beds for COVID-positive newborns in the SNCU.

As reported by gynaecologists, fear of contracting the virus (13; 72%), inaccessibility due to lockdown (7; 39%) and shortage of staff (5; 27.8%) were the major reasons for the decline in services. Half (9; 50%) of the gynaecologists and two-thirds of the paediatricians (12; 70.6%) reported that they faced a shortage of staff; in particular, staff nurses, supporting operation theatre staff and lab technicians. The reported reasons behind the shortage were the diversion of staff for COVID-19-related duties, COVID-19 infection among the staff, and a mandatory quarantine period for staff every fortnight.

#### 3.2.2. Outreach Services including Childhood Immunisation

All DIOs (17; 100%) reported a decline in the number of childhood immunisation sessions during the months of March and April, 2020, as the total number of planned sessions declined considerably during the lockdown period. The major barriers reported by the DIOs in delivering MCH services included the posting of frontline health workers to COVID-19-related duties, inaccessibility to immunisation sites due to lack of transportation facilities during the lockdown, and closure of immunisation session sites.

Out of 108 frontline workers, i.e., ASHA/ANM, the majority (94; 87%) reported that they were posted to COVID-19-related duties, and two-thirds (82; 76%) received training on COVID-19 awareness, its prevention strategies and procedures for conducting rapid antigen tests. More than one-third (42; 39%) of ANMs and ASHAs reported that MCH outreach services provided by them decreased during the lockdown, particularly in May 2020. The major challenges reported by frontline health care workers were related to lack of transportation to visit sub-centres and outreach sessions, COVID-19-related duties, and interrupted supplies of essential drugs and commodities.

## 4. Discussion

This study’s findings documented the barriers faced by patients in accessing maternal and child health services, and the experiences of health care workers in delivering these services during the COVID-19 outbreak period, from March to June, 2020. About two-thirds of the MCH patients did not face any challenges, which may have resulted because the first wave of COVID-19 in India was restricted primarily to urban areas in particularly large metropolitan cities; despite the strict regulations of the first lockdown, the continuum of care in rural areas was maintained.

The challenges reported by the patients in accessing hospital-based services corresponded to the difficulties faced by the health care workers, which were primarily related to a lack of transport facilities, and interrupted hospital-based services both in public and private health care settings. The lack of infrastructural preparedness for outbreak situations and shortages of human resources led to the delayed provision of services. Furthermore, the quarantining of medical staff affected by COVID-19 led to staff shortages at health care facilities. There were no differences noted between red and green zones in the challenges faced by the patients, which indicates that regardless of the severity of spread of COVID-19, the strictness of the mitigation measures may have unnecessarily disrupted MCH services during the nationwide lockdown imposed to prevent and control COVID-19 in India. Furthermore, the multivariable analysis suggested that older women and women who had more than two children had higher odds of experiencing challenges in accessing MCH services during the study period. As reported by health care officials, hospitals providing MCH services were also converted to COVID-19 care units. These measures, along with fear for the little-known COVID-19 infection, resulted in barriers in seeking MCH services [20,21,25]. Similar experiences of challenges faced by patients due to COVID-19 preventive measures have also been reported from other countries [4,14].

The challenges reported by patients corresponded to the difficulties faced by health care workers related to delivery of maternal and child health services at health care facilities. Outreach activities and referrals to health care facilities reduced considerably due to diversion of frontline workers, including ANMs and ASHA workers, to COVID-19-related duties. Studies conducted across the globe during the initial outbreak of the COVID-19 pandemic reported similar observations [15,16,26].

To mitigate challenges associated with the provision of maternal and child health services and impacts in situations of sudden public health emergencies, studies have highlighted the need to explore alternative measures that target community-based MCH services. This approach could prove to be more beneficial than just using a health care-oriented approach in situations of restricted mobility during the lockdown, with a lack of transport to reach health care facilities [27]. The Ministry of Health and Family Welfare, India, announced guidelines such as initiating RMNCAH+N activities in containment zones after 14 days of delisting; using a staggered approach of delivery of services to ensure appropriate COVID-19 preventive measures; limitations to participation in community-based activities; use of PPE by health care providers and front line workers; maintaining an uninterrupted supply of drugs and commodities; home deliveries of essential medicines in containment zones; promotion of tele-consultation services, etc., to be followed across the country [23]. Efforts such as these have been documented in different parts of the globe. A study in Kenya suggested adopting a midwifery model to deliver MCH services during the COVID-19 lockdown in midwifery centres that were located close to the community [28].

### 4.1. Implications to Policy and Practices

Our study has implications for both practice and policy. To minimize the impact of disease outbreaks on maternal and child health care services and future preparedness, the following recommendations may be considered, based on the findings of this study. The recommendations are further categorised on the basis of institutional and outreach services.

#### 4.1.1. Outreach Services

i. Home-based MCH services, with the option of teleconsultation and follow-up by grass-root-level workers (ANM/ASHA). ii. Mobile teams for delivering ANC services and essential medicines in designated containment zones by grass-root-level workers.

#### 4.1.2. Institutional Services

To strengthen hospital-based services, the following recommendations were suggested: i. In the case that a district hospital has been converted into a dedicated COVID-19 hospital, the sub-divisional hospital may be upgraded as the MCH care hospital with a functional SNCU; ii. A dedicated referral mechanism should be in place in each district for emergency MCH services during an epidemic, which identifies and coordinates functional tertiary care centres that deliver MCH services during the outbreak; iii. Public transport during lockdowns may be improved by preparing a plan in consultation with the public transport department and health department, in order to ensure transport facilities for health staff and MCH beneficiaries; iv. Develop a mechanism to ensure the functioning of private health facilities that cater to MCH services during an outbreak.

These recommendations are geared towards effectively strengthening MCH services during a pandemic, and to avert preventable maternal and newborn deaths.

## 5. Strengths and Limitations

The strength of the study is that it provides perspectives from both patients and health care providers from six states of India. The study’s findings need to be considered in light of several limitations, such as conducting telephone interviews which have limitations such as short interview durations; the absence of visual or nonverbal cues that cannot be equated with face-to-face interviews; and recall bias, considering the period of data collection. The study primarily focused on challenges faced by patients who availed MCH services, and may have ignored the challenges faced by patients who were not able to access services. The list of patients was randomly selected from the records of frontline workers, in order to minimise selection bias; however, the possibility of some inherent selection bias in the sampling strategy cannot be ignored.

## 6. Conclusions

The COVID-19 pandemic disrupted routine MCH services in the country. The challenges faced by the MCH patients were primarily a lack of transportation, and difficulties in accessing hospital-based services due to inadequate human resources and infrastructure. In order to maintain MCH services during a pandemic, a holistic approach is required which focuses on both preparedness and response to the outbreak, as well reassignment and reinforcement of health care professionals to cater to essential MCH services.

## Figures and Tables

**Table 1:** Sociodemographic profile of the patients (n = 540) from six states of India, March–June 2020.

Sociodemographic Characteristics (n = 540)	No. of Women	Percent
**Age in years**		
15–19	35	6.5
20–24	256	47.4
25–29	179	33.1
30–34	54	10.0
≥35	16	3.0
**Education (years of schooling)**		
No schooling	37	6.9
Less than 5 years	13	2.4
5–7	55	10.2
8–9	104	19.3
10–11	137	25.4
12 or more years	194	35.9
**Religion**		
Hindu	450	83.3
Muslim	72	13.3
Others	18	3.3
**Number of children**		
No child	64	11.9
1	250	46.3
2	160	29.6
More than 2	66	12.2
**Having ration card**		
Yes	319	59.1
No	221	40.9
**Main Source of Income**		
Agriculture	156	28.9
Agricultural wages/Non-agricultural wages	122	22.6
Small Business	103	19.1
Salaried Employment/Professional	130	24.1
Pension/Rent/Dividend/Others	29	5.4
**Main Source of Income During Lockdown**		
No Income	307	56.9
Government Schemes	12	2.2
Agriculture	104	19.3
Skilled/Unskilled labour	31	5.7
Small Business	24	4.4
Salaried Employment/Professional	40	7.4
Pension/Rent/Dividend/Others	22	4.1
**COVID zone**		
Red	361	66.9
Green	179	33.1
Total	540	100

**Table 2:** Unadjusted and adjusted odds ratios and confidence intervals with respect to possible risk factors related to the challenges faced by the patients in accessing MCH services during lockdown.

Socio-Demographic Characteristics	Unadjusted Odds Ratio (OR)				Adjusted Odds Ratio (OR)			
	Exp(B)	Sig.	95% C.I. for Exp(B)		Exp(B)	Sig.	95% C.I. for Exp(B)	
			Lower	Upper			Lower	Upper
**COVID zone**								
Red								
Green	0.89	0.54	0.62	1.29	0.88	0.518	0.59	1.31
***Age***								
15–24								
25 and above	1.18	0.35	0.83	1.67	1.57	0.025	1.06	2.32
**Education**								
No schooling and less than 5 years								
5–7	1.53	0.30	0.69	3.40	1.42	0.402	0.62	3.24
8–9	1.12	0.75	0.55	2.31	0.99	0.969	0.46	2.09
10–11	1.76	0.11	0.89	3.48	1.87	0.092	0.9	3.88
12 or more years	1.34	0.039	0.69	2.59	1.36	0.405	0.66	2.78
**Religion**								
Hindu								
Muslim	1.68	0.04	1.02	2.77	1.97	0.014	1.14	3.38
Others	1.34	0.54	0.052	3.47	1.3	0.597	0.49	3.45
**Number of children**								
No child								
1	0.83	0.51	0.48	1.45	0.81	0.474	0.45	1.45
2	0.76	0.37	0.42	1.37	0.68	0.232	0.36	1.28
More than 2	0.49	0.05	0.24	1.01	0.36	0.014	0.16	0.81
**Having ration card**								
No								
Yes	0.99	0.97	0.70	1.41	1.02	0.935	0.7	1.47
**Main Source of Income**								
Agriculture								
Agricultural wages/Non-agricultural wages	1.27	0.33	0.79	2.06	1.5	0.151	0.86	2.62
Salaried Employment/Professional	0.88	0.59	0.54	1.42	0.91	0.747	0.52	1.61
Small Business/Pension/ Rent/Dividend/Others	1.04	0.87	0.65	1.67	0.96	0.882	0.56	1.66
**Main Source of Income During Lockdown**								
No Income								
Agriculture	1.11	0.64	0.71	1.75	1.28	0.362	0.75	2.19
Other	0.84	0.42	0.55	1.29	0.89	0.62	0.57	1.39

## Data Availability

The datasets generated and/or analysed during the current study are not publicly available, as we are a Government of India research organisation; approvals from the competent authority need to be taken for data sharing, but the data may be available from the corresponding author on reasonable request, subject to approval from the competent authority.

## Abbreviations

MCH	Maternal and Child Health
DIO	District Immunisation Officer
ASHA	Accredited Social Health Activist
ANM	Auxiliary Nurse Midwife
KCR	K Chandrashekar Rao kit
SNCU	Special Newborn Care Unit
